# A Study on Hematopoietic Stem Cell Donation Volunteer Retention between Swab Sampling Approach and Blood Sampling Approach: Evidence from Shanghai, China

**DOI:** 10.3390/ijerph18084027

**Published:** 2021-04-12

**Authors:** Ke Yan, Gang Zhang, Guoqiang Zhao, Baosong Liu, Jun Lu

**Affiliations:** 1 Department of Health Policy and Management, School of Public Health, Fudan University, Shanghai 200032, China; 18111020031@fudan.edu.cn (K.Y.); 15211020034@fudan.edu.cn (B.L.); 2 China Research Center on Disability Issues, Fudan University, Shanghai 200032, China; 3 Key Laboratory of Health Technology Assessment, National Health Commission, Fudan University, Shanghai 200032, China; 4 Red Cross Society of China Shanghai Branch, Shanghai 200040, China; ZG1465@163.com (G.Z.); sonics8162@126.com (G.Z.)

**Keywords:** hematopoietic stem cell donation, sampling approach, volunteer retention

## Abstract

The loss of hematopoietic stem cell donation (HSCD) volunteers is widespread worldwide. This study analyzed the distribution characteristics of volunteer retention between the swab sampling approach and blood sampling approach. The Shanghai branch of the China Bone Marrow Donation Program conducted a telephone follow-up with 18,963 volunteers to understand volunteer retention. Multiple logistic regression was used to analyze the distribution characteristics of volunteer retention between two different sampling approaches, and a forest plot was used to observe the distribution trend. Only 32.37% of the volunteers could be contacted, and the loss of volunteers was severe. The volunteer retention is influenced by sampling approaches and demographic characteristics, and Shanghai natives, the highly educated, and students had better retention. The volunteer retention of the swab group was better among young people and technicians, while the volunteer retention of the blood sample group was lower among public officials and workers, and the volunteer retention in the blood sample group was more significantly affected by changes in population characteristics. To enhance the stability of volunteers, managers should improve the contact channels and frequency, expand the ratio of stable volunteers, strengthen volunteer education in the process of collecting blood samples, and respect individuals’ willingness.

## 1. Introduction

Hematopoietic stem cell transplantation (HSCT) is one of the most effective methods to treat hematologic malignancies [1]. Two-thirds of patients with hematopoietic diseases rely on non-related donors to donate hematopoietic stem cells [2], although the match rate of the human leukocyte antigen (HLA) of non-blood-related people is only 1/400 to 1/10,000 [3]. To save leukemia patients, countries worldwide have implemented marrow donor programs, responsible for the publicity, organization, mobilization, sampling, HLA typing, matching, and donations of voluntary hematopoietic stem cell donors.

The National Marrow Donor Program in the United States and Zentrales Knochenmarkspender-Register Deutschlandde in Germany have two regular sampling approaches: one is to collect 6–8 mL of venous blood from volunteers by medical staff for testing and HLA typing; the other is that volunteers apply for a swab sampling tool online, scrape the oral mucosa sample by themselves, and then mail it to the testing center [4,5].

The Chinese Marrow Donor Program (CMDP) has always collected blood samples for HLA typing. Moreover, the pilot for collecting hematopoietic stem cell samples through swab samples only began in 2015 to diversify the way volunteers can join the CMDP. This approach is convenient, rapid, and non-invasive and does not require specialized medical personnel [6]. One of the first cities to conduct pilot applications was Shanghai.

The process, from volunteer registration to donation of hematopoietic stem cells, is long, ranging from several months to decades. During this period, the hematopoietic stem cell bank maintains good contact with existing volunteers so that the volunteers can be contacted smoothly when patients request donations. This is an important condition for the completion of HSCT [7]. However, the loss of contact between hematopoietic stem cell banks and volunteers is a worldwide phenomenon.

The World Marrow Donor Association (WMDA) report showed that the loss of volunteers occurs in different countries and regions [8]. In 2019, the CMDP received 9759 retrieval applications from home and abroad. While the success rate of the initial HLA matching was 95.19%, the success rate of high HLA matching was only 15.30% [9]. The failure to contact volunteers after the initial HLA matching was one of the most important influencing factors [10,11]. It was reported that a patient found seven HLA-matched volunteers after the initial HLA match, but was only able to contact two of them, a successful contact rate of only 28.57%. Finally, the patient found a suitable donor among the two volunteers. However, in reality, many patients have lost their chance of survival while waiting because they failed to contact the volunteers [12,13]. The low volunteer retention rate reduces the number of effective volunteers and the success rate of patient matching, which limits the functional value of the Marrow Donor Program.

Many researchers have realized the seriousness of this problem. Aljurf et al. [14] analyzed the WMDA report and found that reasons for the loss of volunteers included lack of awareness, region, race, and religion, with the donation rate of the white population in the US being 30% higher than that of other races. Moreover, some ethnic minorities have lower trust in stem cell transplantation due to religious and cultural beliefs. Beom et al. [15] suggested that the Korean Network for Organ Sharing Database strengthens the contact management of volunteers while actively recruiting new volunteers to ensure the effectiveness of volunteers and improve the cost-effectiveness of the Hematopoietic Stem Cell Bank. Evseeva et al. [8] suggested that maintaining good communication with volunteers can help in contacting volunteers quickly and reduce the waiting time for patients to receive transplants. These studies have helped confirm the significance of increased volunteer retention, finding differences between countries and regions; however, no studies have discussed the relationship between volunteer retention and sampling approaches.

Our study analyzed the retention of 18,963 volunteers in the Shanghai branch of the CMDP. According to different sampling approaches, volunteers were divided into the swab sample group and blood sample group, and the retention distribution characteristics of the two groups of volunteers were analyzed. This study will help researchers and policymakers understand the application characteristics of the two sampling approaches and the appropriate populations, to make targeted interventions to increase the retention rate of hematopoietic stem cell donation (HSCD) volunteers and promote the development of HSCD.

## 2. Materials and Methods

### 2.1. Data Source and Study Design

From 2016 to 2017, the Shanghai branch of the CMDP conducted a sample telephone follow-up for the volunteers recruited from 2013–2017 and conducted uniform training for investigators to ensure the consistency of the follow-up process and standards. A total of 18,963 volunteers were interviewed via telephone, including 5792 who had swab samples taken and 13,171 who had blood samples taken. The sampling approach between 2013 and 2014 was only blood, while the swab sampling approach was added from 2015. The blood sampling approach is when medical staff extract the venous blood of the volunteers in a fixed place for HLA typing detection, while the swab sampling approach allows volunteers to scrape their oral mucosal tissues anywhere with the sampling tool applied for online to collect samples for HLA typing.

**Figure 1:**
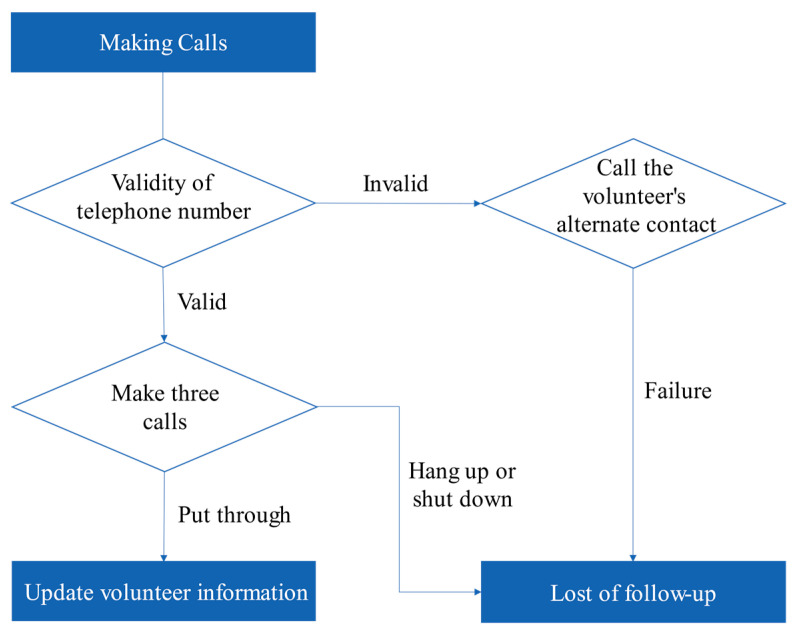
Telephone follow-up flow chart.

During the telephone follow-up, the volunteers or their alternate contacts were dialed at three different times. If one of the following situations occurred, it was recorded that the volunteer was lost to follow-up: the reserved phone number was invalid, and three consecutive calls were hung up or shut down. If the volunteers were successfully contacted, the personal information and contact information was updated, the volunteers was given information and education, and the volunteers were thanked for their dedication. Volunteers who showed unsatisfactory willingness to donate during the return visit were re-mobilized and received follow-up return visits. The follow-up process is shown in Figure 1.

### 2.2. Variable Definitions

According to the different sampling approaches, we divided the volunteers into the swab sample and blood sample group, observed the return visits of the two groups of volunteers, and analyzed the distribution characteristics of volunteer retention.

The volunteers’ telephone follow-up results were dependent variables, including volunteer retention and volunteers lost to follow-up. Taking the remaining volunteers as the reference group, the influencing factors of the loss to follow-up were analyzed.

According to the population characteristics, gender was divided into male and female; ethnic group into Han ethnicity and ethnic minorities; census registration into Shanghai native and non-natives; the age groups into <25, 25–34, 35–44, and ≥45 years; educational levels into middle school, college, and postgraduate; occupations into students, technicians, public officials, workers, and others; the frequency of blood donation into 0 times, 1 time, and multiple times; and the registration time referring to the time from the volunteer joining the CMDP to the follow-up, including 3 years, 2 years, and 1 year.

### 2.3. Data Analyses

Descriptive analysis and the Pearson chi-square test were used to describe the population characteristics of the HSCD volunteers. Charts were generated using Microsoft Excel to show the overall results of the volunteers’ follow-up. Multiple logistic regression was used to analyze the difference in the distribution of volunteer retention between the swab group and the blood group and to explore the influencing factors. The forest plot was used to observe the distribution trend of volunteer retention between the two groups. The *p* values were two-sided, and *p* < 0.05 was considered statistically significant. All analyses were performed using SPSS 22.0 (SPSS Inc., Chicago, IL, USA).

## 3. Results

Of the 18,963 volunteers in the present study, there were more men (54.75%) than women (Table 1). In China, the Han ethnicity accounts for the vast majority of the population. In this study, the Han ethnicity accounted for 98.17%. Shanghai has a large non-native population, and volunteers from this population accounted for approximately 70%. Volunteers are mainly young people, with 73.88% of those younger than 35 years old, with the average age of the overall sample (±standard deviation) being 29.65 ± 7.53 years. Volunteers had a higher level of education, with college degree-holding volunteers accounting for 60.55%. In the occupational classification, the student population exceeded 1/3. Blood donation experience was found in 32.95% of the volunteers, while 14.91% of the volunteers had donated blood many times. Three-quarters of the volunteers registered within the previous year. There were statistically significant differences in the distribution of population characteristics between the swab sample group and blood sample group (*p* < 0.05).

**Figure 2:**
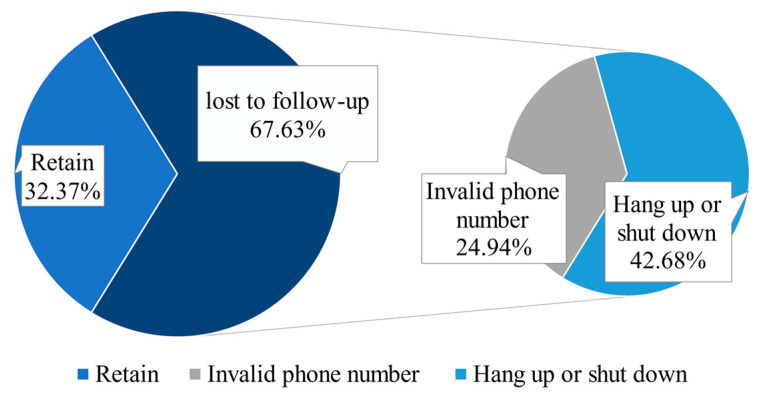
Telephone follow-up results of HSCD volunteers.

In Figure 2, in the telephone follow-up, only 32.37% of the volunteers were successfully contacted, less than 1/3 of the total number, and the loss was severe. Of the 67.63% of the volunteers who were lost to follow-up, 42.86% of them hung up or shut down the return call, and 24.94% of the volunteers’ phone numbers were invalid.

**Figure 3:**
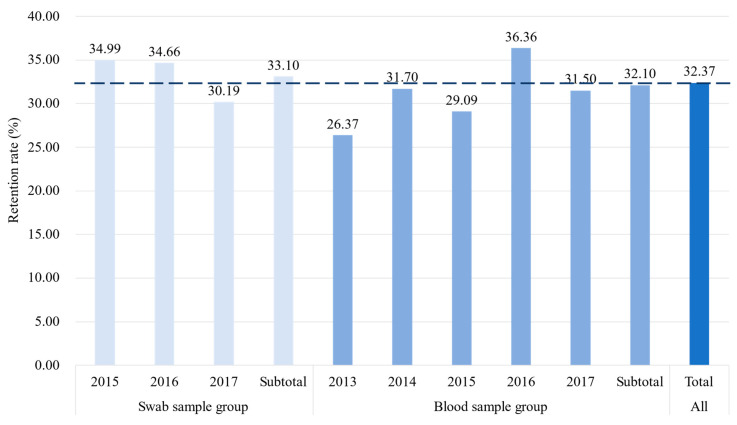
HSCD volunteer retention rate by registration year.

Figure 3 shows the retention rate of volunteers registered from 2013 to 2017. The dotted line in the figure represents an average retention rate of 32.27% of volunteers. The retention rate in the swab sample group varied slightly from 30.19% to 34.99%, with an average retention rate of 33.10%. The retention rate of volunteers in the blood sample group fluctuated greatly, with the lowest (26.37%) in 2013 and the highest (36.36%) in 2016, and the average retention rate was 32.10%. The retention rate of volunteers in the swab sample group is slightly higher than that in the blood sample group, and the retention rate is also more stable.

In Table 2, Model 1 analyzes the data of all volunteers from 2013 to 2017, and Model 2 analyzes the data of volunteers recruited from 2015 to 2017. Adjusting for population characteristics in Model 1 and Model 2, we found statistically significant differences in volunteer retention between the swab sample group and the blood sample group (*p* < 0.001). It is necessary to further explore the appropriate population of the two sampling approaches.

According to the different sampling approaches, we established Model 3 and Model 4 in Table 3, analyzing the association between the volunteer retention and population characteristics of the swab sample group and blood sample group, respectively. In these two models, we found that Shanghai native volunteers (*p* < 0.05) had better retention than the non-native population, and the volunteer retention of those over the age of 35 years or those with high education or students was also better (*p* < 0.05).

The distribution of volunteer retention in the population of the two groups also showed some differences. In the swab sample group, volunteers aged 25–34 years (odds ratio (OR) = 0.672, *p* < 0.001) or technicians were well retained (OR = 0.743, *p* = 0.005).

In the blood sample group, there was a high loss of volunteers who were public officials (OR = 1.193, *p* = 0.029) or workers (OR = 1.308, *p* = 0.001). The retention of volunteers who had donated blood several times was better (OR = 0.844, *p* = 0.002). With increased time from registration, the outflow of volunteers increased gradually (registration time of 2 years (OR = 1.101, *p* < 0.001) and registration time of 3 years (OR = 1.529, *p* < 0.001).

**Figure 4:**
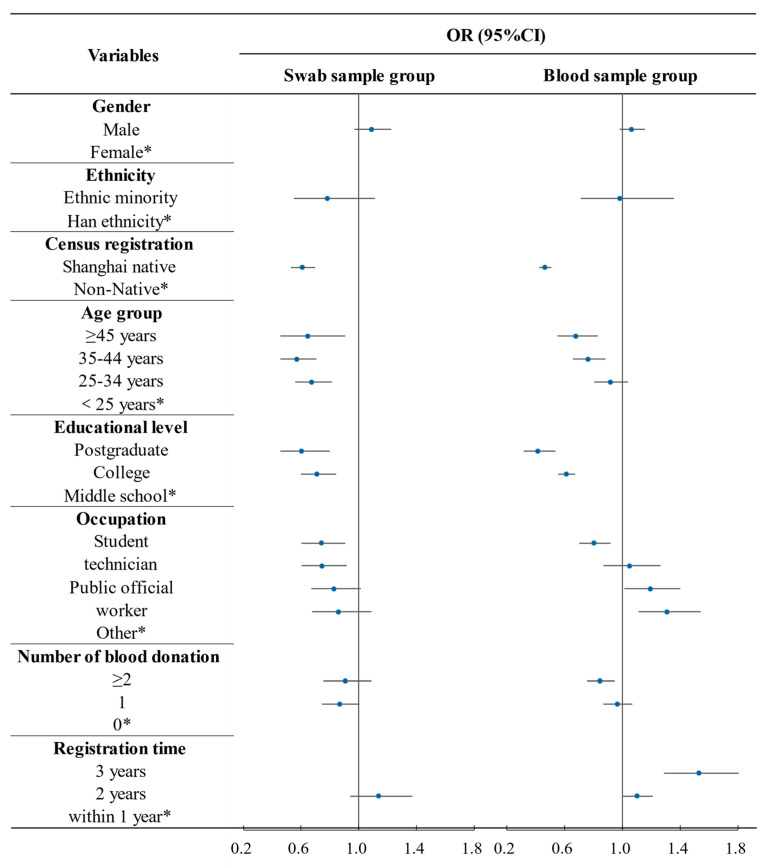
Forest plot of volunteer retention in the swab sample group and blood sample group. OR: odds ratio. CI: confidence interval. The origin in the forest plot represents the OR value, and the horizontal line represents 95% CI. * Indicates a reference.

Figure 4 shows the distribution trend of volunteer retention in the population. In general, the OR value in the swab sample group always changed slightly around the value 1, and the OR value of the blood sample group had a large range of changes and a higher degree of dispersion. With the change of population characteristics, the change trend of volunteer retention in the blood sample group was more obvious than that in the swab sample group. In the blood sample group, volunteer retention increased step by step with age, education level, or number of blood donations. However, with increased time from registration, volunteer retention decreased rapidly. At the same time, the change trend of volunteer retention among different household registrations or occupations was also more obvious than that of the swab sample group.

## 4. Discussion

The capacity of the Shanghai branch of the CMDP has been increasing yearly. As of 2019, there were 147,315 volunteer donors [9]. It is important to maintain a large database of volunteer donors. However, in the telephone follow-up in this study, only 32.37% of the volunteers were contacted successfully, and the loss of volunteer donors was severe. With an increase in the number of telemarketing and scam telephone calls, people have become unwilling to answer unfamiliar calls. Research reported that the response rate in a nationwide random telephone survey of the Social Opinion Survey Center of the National Bureau of Statistics of China was 10–20% [16]. The telephone survey response rate of the Pew Research Center in the United States was 9% [17]. In this case, the CMDP can increase trust by using iconic numbers such as those used by fire departments and emergency centers.

There are some similarities in the distribution characteristics of volunteer retention between the swab sampling approach and blood sampling approach. First, the retention of the migrant population was lower than that of the Shanghai registered population. Shanghai is an international metropolis with a large number of young and middle-aged people from elsewhere [18]. The migrant volunteers in the sample of this study accounted for 69.11%, but their mobility is very high. Many people leave Shanghai due to the completion of their studies, job changes, family relocation, and other reasons [19,20]. They change their contact information or think it is unnecessary to answer calls from Shanghai. We also observed a high cutoff among volunteers under the age of 25 years, which may also be associated with greater mobility among young people [21]. This suggests that the recruitment ratio of Shanghai natives can be increased to improve the stability of volunteers. At the same time, in addition to telephone contact, the application of social networking such as e-mail, WeChat, and Twitter should be strengthened [22].

Second, highly educated people and students are also potential groups of stable volunteers. This is in line with the current social phenomenon: in large-scale social surveys, respondents with a high level of education were more likely to be found and more willing to participate in the survey [23,24]. The retention of student volunteers was better because they tended to be more enthusiastic about public welfare [25].

In addition, the distributions of volunteer retention characteristics of the two groups had their own characteristics. Volunteer retention in the swab sample group was less affected by population characteristics than that in the blood sample group. In the swab sample group, people can learn about relevant information through online news, social media, etc., and then apply for swab sampling tools online, scrape the oral mucosal swab samples by themselves, and mail them to the testing center. This process takes about a week, and the volunteers have a relatively long time for serious consideration. Therefore, the volunteer retention varies slightly among different population characteristics.

In the blood sample group, the volunteer retention varied significantly with demographic characteristics. The blood sample collection methods are mostly used when organizing medical staff at government departments, enterprises, and public places, and to carry out volunteer publicity, mobilization, and sampling for groups. During this process, more emphasis was placed on the importance of charitable acts with a lack of explanation of HSCT, and the understanding of volunteers’ subjective wishes was also insufficient. Some volunteers joined the CMDP out of temporary enthusiasm, or some even for subsidies or vacations, which led to a large difference in volunteer retention among different groups of people with different characteristics, because these characteristics reflect the differences in the volunteers’ cognition, experience, thoughts, and environment [26,27].

Meanwhile, there were differences in the distribution of volunteer retention rates between the groups regarding age, occupation, blood donation frequency, and registration time. In view of the different levels of awareness and acceptance of new technologies, the volunteer retention in the swab sample group is better among young people aged 25–34 and technicians. Over-recruitment of volunteers in government units and large enterprises may be the reason for the poor retention of public officials and worker volunteers in the blood sample group. Concurrently, due to inadequate publicity and education in the collective recruitment activities, volunteers in the blood sample group often lack in-depth thinking when they join the bone marrow bank, causing the loss of volunteers to increase significantly as the time from registration increases. We also found that in the blood sample group, volunteers who had donated blood frequently had better retention. Therefore, the swab sampling approach can strengthen the promotion among young people and technical personnel. In the collective recruitment of volunteers through the blood sampling approach, it is necessary to strengthen the propaganda and education of the volunteer groups, respect the personal wishes of volunteers, strengthen the frequency of contact with them, and mobilize people who have had several blood donation experiences to retain blood samples for HLA typing [28].

### Limitations

There were some limitations in this study. First, due to the large number of volunteers, only some of the volunteers were interviewed according to the convenience sampling, which may have led to sample selection bias. Second, this study is an observational study, not a controlled trial. Therefore, some confounding variables cannot be controlled, such as volunteers’ physical, family, and economic condition, which may affect the accuracy of statistical analysis results. Finally, all the volunteers in this study joined the bone marrow bank after 2013, and the time from registration was relatively short. The follow-up of volunteers registered before 2013 is still underway, and further research is recommended.

## 5. Conclusions

The results of the study showed that the HSCD volunteer retention was low and the loss was severe. Measures should be taken to diversify contact channels and applied for specific landmark numbers in order to maintain good communication with volunteers.

The volunteer retention was influenced by sampling approaches and population characteristics. According to the distribution characteristics of volunteer retention, managers should expand the recruitment ratio of stable volunteers, increase the proportion of Shanghai natives, higher education population, and student population, promote the application of swab sampling approach among young people and technical personnel, and mobilize people who have had several blood donation experiences to retain blood samples for HLA typing. Meanwhile, targeted measures should be taken to improve the recruitment process, strengthen the publicity and education of volunteers during the collective collection of blood samples, and fully respect the personal wishes of volunteers, so as to enhance the stability of volunteers.

## Figures and Tables

**Table 1:** General population characteristics of the hematopoietic stem cell donation (HSCD) volunteers according to sampling approach.

Variables		Total	Sampling Approach		
			Swab ^a^	Blood ^b^	*p* ^c^
		N = 18,963	N = 5792	N = 13,171	
		%	%	%	
Sex	Male	54.75	13.04	41.72	*p* < 0.000 **
	Female	45.25	17.51	27.74	
Ethnicity	Ethnic minority	98.17	29.73	68.44	*p* < 0.000 **
	Han ethnicity	1.83	0.82	1.01	
Census registration	Shanghai native	30.89	10.63	20.27	*p* < 0.000 **
	Non-native	69.11	19.92	49.19	
Age group	<25 years	33.80	12.78	21.02	*p* < 0.000 **
	25–34 years	40.08	10.59	29.48	
	35–44 years	21.29	5.97	15.32	
	≥45 years	4.83	1.20	3.63	
Educational level	Middle school	36.10	6.63	29.47	*p* < 0.000 **
	College	60.55	22.09	38.47	
	Postgraduate	3.35	1.83	1.52	
Occupation	Student	34.59	13.69	20.90	*p* < 0.000 **
	Technician	6.37	3.32	3.05	
	Public official	7.80	3.28	4.52	
	Worker	8.03	2.49	5.54	
	Other	43.21	7.76	35.44	
Number of blood donation	0	67.05	21.42	45.63	*p* < 0.000 **
	1	18.04	5.48	12.56	
	≥2	14.91	3.64	11.27	
Registration time	Within 1 year	75.05	27.03	48.02	*p* < 0.000 **
	2 years	20.63	3.51	17.12	
	3 years	4.32	0.00	4.32	

Note. ^a^ The swab sampling approach was applied from 2015 to 2017. ^b^ The blood sampling approach was applied from 2013 to 2017. ^c^ *p* values were calculated using the chi-square test for categorical variables. ** *p* < 0.001.

**Table 2:** Effect of sampling approach on volunteer retention.

Variables		Model 1 ^a^ (All Volunteers)		Model 2 ^b^ (Volunteers Registered from 2015–2017)	
		OR	*p*	OR	*p*
Sampling approach	Swab	1.157	<0.001 **	1.159	<0.001 **
	Blood	Reference			

Note: ^a^ Model 1 analyzed the data of all volunteers recruited from 2013 to 2017. ^b^ Model 2 analyzed the data of volunteers recruited from 2015 to 2017. In Model 1 and Model 2, multiple logistic regression was used to analyze the retention of volunteers between the two sampling methods, and the population characteristics were adjusted. ** *p* < 0.001.

**Table 3:** Multiple logistic regression model of volunteer retention according to sampling approaches.

Variables		Model 3 ^a^ (Swab Sample Group)		Model 4 ^b^ (Blood Sample Group)	
		OR	*p*	OR	*p*
Sex	Male	1.087	0.153	1.062	0.141
	Female	Reference			
Ethnicity	Ethnic minority	0.781	0.164	0.981	0.907
	Han ethnicity	Reference			
Census registration	Shanghai native	0.607	0.000 **	0.462	0.000 **
	Non-native	Reference			
Age group	≥45 years	0.645	0.011 *	0.676	0.000 **
	35–44 years	0.569	0.000 **	0.761	0.000 **
	25–34 years	0.672	0.000 **	0.916	0.170
	<25 years	Reference			
Educational level	Postgraduate	0.602	0.000 **	0.413	0.000 **
	College	0.708	0.000 **	0.611	0.000 **
	Middle school	Reference			
Occupation	Student	0.740	0.003 *	0.802	0.001 *
	Technician	0.743	0.005 *	1.049	0.612
	Public official	0.826	0.065	1.193	0.029 *
	Worker	0.857	0.195	1.308	0.001 *
	Other	Reference			
Number of blood donations	≥2	0.905	0.282	0.844	0.002 *
	1	0.866	0.055	0.964	0.474
	0	Reference			
Registration time	2 years	1.136	0.177	1.101	0.000 **
	Within 1 year	Reference			

Note: ^a^ Model 3 analyzed the data of volunteers recruited through swab sampling approach. ^b^ Model 4 analyzed the data of volunteers recruited through blood sampling approach. Model 3 and Model 4 both performed multiple logistic regression. ** *p* < 0.001. * *p* < 0.05.

## Data Availability

The data are not publicly available to protect the confidentiality of the participants.
